# Aggregation-Prone Proteins Modulate Huntingtin Inclusion Body Formation in Yeast

**DOI:** 10.1371/currents.hd.501008f3051342c9a5c0cd0f3a5bf3a4

**Published:** 2014-04-23

**Authors:** Ralitsa B. Kantcheva, Robert Mason, Flaviano Giorgini

**Affiliations:** Department of Genetics, University of Leicester, Leicester, UK; Department of Genetics, University of Leicester, Leicester, UK; Department of Genetics, University of Leicester, Leicester, UK

## Abstract

Huntington's disease (HD) is a fatal neurodegenerative disorder caused by a polyglutamine expansion in the huntingtin (HTT) protein. The expression of mutant HTT in the baker’s yeast Saccharomyces cerevisiae recapitulates many of the cellular phenotypes observed in mammalian HD models. Mutant HTT aggregation and toxicity in yeast is influenced by the presence of the Rnq1p and Sup35p prions, as well as other glutamine/asparagine-rich aggregation-prone proteins. Here we investigated the ability of a subset of these proteins to modulate mutant HTT aggregation and to substitute for the prion form of Rnq1p. We find that overexpression of either the putative prion Ybr016wp or the Sup35p prion restores aggregation of mutant HTT in yeast cells lacking the Rnq1p prion. These results indicate that an interchangeable suite of aggregation-prone proteins regulates mutant HTT aggregation dynamics in yeast, which may have implications for mutant HTT aggregation in human cells.

## Introduction

Huntington’s disease (HD) is an autosomal dominant neurodegenerative disease 1 ^,^ 2 caused by the expansion of a polyglutamine (polyQ) tract in the huntingtin (HTT) protein, which leads to its misfolding and aggregation^3^ . Intracellular HTT aggregates are a pathological hallmark of HD^4^^,^ 5, with their morphology and localization influencing downstream cellular effects^6^^,^ 7 . Several studies suggest that macromolecular HTT aggregates which prevent further intermolecular interactions are neuroprotective^8^, while soluble oligomeric species of mutant HTT capable of sequestering other cellular proteins are neurotoxic^9^ . HD has been modelled in a wide range of organisms^10^^,^ 11 including the baker’s yeast, *Saccharomyces cerevisiae^12^*. Yeast models recapitulate many of the cellular disease phenotypes seen in HD patients, including disturbances in mitochondrial function, increased production of reactive oxygen species, disruption of the cytoskeleton, proteasome dysfunction and increased flux through the kynurenine pathway of tryptophan degradation^13^ .

A number of yeast models have been developed to study the cellular effects of mutant HTT expression^7^^,^ 12 ^,^ 14 . These models have shown that aggregation and cellular toxicity of mutant HTT in yeast is dependent upon the presence of the Rnq1p in its prion [*PIN^+^*] conformation^14^ , as deletion of *RNQ1* or curing of [*PIN^+^*] ameliorates these phenotypes. Furthermore, a network of prion-like glutamine-rich proteins modulates mutant HTT-dependent toxicity in yeast^15^ , with expression of these proteins - as well as Rnq1p - having an additive effect on the overall level of toxicity observed. Although the cellular function of Rnq1p is unclear, it is crucial for the *de novo* formation of [*PSI^+^*], the prion form of Sup35p^16^^,^ 17 . [*PSI^+^*] is one of the most extensively studied yeast prions and is encoded by *SUP35*, with the non-prion form of the protein being crucial for the release of newly synthesized polypeptides from the ribosomal complex through ATP hydrolysis^18^ . A recent study found that Sup35p interacts with mutant HTT through its Q/N-rich prion domain, and the presence of [*PSI^+^*] is critical for full toxicity of mutant HTT constructs with extended polyproline regions^19^ .

We previously identified a number of putative yeast prions in a screen for gene deletion suppressors of HTT103Q-dependent toxicity in yeast^20^ . The proteins in question - Def1p, Ybr016wp, Yir003wp and Ylr278cp - all have glutamine/asparagine-rich (Q/N-rich) domains and computational analyses suggests that they share primary sequence features with known prions^21^ . Subsequent work has found that Def1p, Yir003wp and Ylr278cp do not behave as typical yeast prion proteins, while Ybr016wp has a large number of characteristics in common with established yeast prions such as Sup35p^22^ . Here we further investigate the effect of Ybr016wp and Sup35p on mutant HTT aggregation dynamics in yeast. In order to unmask the effect these proteins have on this protein misfolding process, we focused our attention on changes in HTT aggregation dynamics in a *rnq1*∆ background. We find that both Sup35p and Ybr016wp modulate the formation of mutant HTT aggregates in yeast and that these effects are partially dependent on the presence of Rnq1p in the cell. In total, this work indicates that other aggregation-prone proteins can substitute for Rnq1p in the context of HTT misfolding, and suggests that dynamic interplay between a suite of such proteins modulates HTT aggregation in yeast, which may have implications for HTT aggregation in human cells.

## Methods

**Yeast strains and culturing: **The BY4741 and *rnq1*Δ deletion strains (MAT a*, *his3Δ*1, *leu2-3,112, trp1-1, ura3-1, ade2-1****) employed carry integrated constructs encoding a human HTT fragment consisting of the first 17 N-terminal amino acids followed by either 25 or 103 glutamines^7^ . Yeast strains were typically grown on YPD (1 % yeast extract, 2 % peptone, 2 % glucose, 2.5 % agar). Recombinant strains were selected on synthetic medium lacking relevant amino acids (0.68 % yeast nitrogen base without amino acids, 2.5 % agar, 2 % glucose, 0.6 % leucine, 0.3 % lysine, 0.09 % dropout powder lacking the respective amino acids^23^ ).

**Yeast growth assays: **Yeast colonies were inoculated into 100 µl of medium in 96-well plates and incubated at 30 ºC for 16 to 18 hours. Cultures were serially diluted by 5 or 10-fold in distilled water and spotted (5 μl) onto selective plates containing either 2 % galactose or 2 % glucose as a carbon source and incubated for 60 to 72 hours at 30 ºC.

**Fluorescence microscopy** **: **Colonies were inoculated in 5 ml of glucose containing selective medium and incubated at 30 ºC with shaking for 24 hours. Cultures were washed once with water and diluted to an OD_600 _of 0.2 in 5 ml selective media containing 2 % raffinose. After a further 6 hours at 30 ºC with shaking, galactose was added to a final concentration of 2 % to induce expression of constructs under the control of *GAL1 *promoters. After a further 12 hours the number of cells containing aggregates was scored using a Zeiss Axioscope 2 fluorescent microscope with a Zeiss 100 X Plan-NEOFLUAR (1.30/oil, ∞/0.17) objective and a chromomycin filter.

**Determination of yeast *[PIN]* and *[PSI]* status: **The presence of the [*PIN^+^*] prion was detected by a previously described protocol^24^ using a primary antibody against Rnq1p (Santa Cruz Biotechnology Inc., 1:10,000 dilution). Samples for the determination of [*PSI*] status were grown the same way as for the [*PIN*] status assay, and 4 OD units harvested by centrifugation and suspended in 100 µl of ice-cold lysis buffer [1X PBS without Ca^2+^and Mg^2+^(PAA Laboratories GmbH, Austria), 100 mM NaCl, 2 mM PMSF and 1x EDTA-free protease inhibitors (Roche)]. Samples were lysed with 100 µl of acid washed glass beads (425-600 μm, Sigma-Aldrich, USA) in a bead-beater for 1 minute at maximum speed. After adding an additional 100 µl of ice-cold lysis buffer, samples were left on ice for 1 minute to allow the beads to sediment and the supernatant was removed for further analysis. The amount of protein was quantified using a NanoPhotometer™ Pearl (IMPLEN GmbH, Germany) and 10 μg of protein in 50 μl of lysis buffer were spun at 50,000 rpm at 4 ºC for 15 minutes in a BECKMAN TL-100 ultracentrifuge (Beckman, USA). The supernatant was removed and used as the “Soluble” fraction, while the pellet was resuspended in 50 μl of lysis buffer and used as the “Pellet” fraction. Protein from the total, soluble and pellet fractions were mixed with 2 µl of 5 X protein loading dye [50 mM Tris-HCl (pH 6.8), 2 % SDS, 10 % glycerol, 1 % β-mercaptoethanol, 12.5 mM EDTA, and 0.02 % bromophenol blue] to give a final volume of 10 μl. Samples were denatured at 95 ºC for 5 minutes, separated by SDS-PAGE, and transferred to PVDF membranes. Sup35p was detected with a primary antibody against yeast Sup35p (1:10,000 dilution), a generous gift from Mick Tuite (University of Kent, UK).

**Statistical Analyses: **Data was analysed by unpaired, two-tailed Mann-Whitney tests using Prism 6 (GraphPad Software).

## Results

**HD model yeast exhibit HTT aggregation independent of toxicity in a [*PIN^+^*] and [*PSI^+^*] background: **To avoid confounding issues due to variable plasmid copy number we generated yeast strains containing either HTT25Q or HTT103Q constructs integrated at the *HIS3 *locus in parental BY4741 yeast, as well as in a *rnq1*Δ strain in the same background. The Rnq1p-deficient strain was generated by deleting *RNQ1 *using homologous recombination, which was verified by PCR genotyping and immunoblotting (Figure 1b; data not shown). These constructs encode the first 17 amino acids of HTT followed by the respective polyQ length under the control of an inducible *GAL1 *promoter, and have an N-terminal FLAG epitope tag and a C-terminal CFP fusion. Integration of the HTT constructs was achieved by employing the integrative vector pRS303, and successful integration events were confirmed by PCR and sequencing of the *HIS3 *locus (data not shown).

**An integrated HD yeast model exhibits a reduced level of mutant HTT toxicity in the cell independent of the presence of [PIN+] and [PSI+]:**
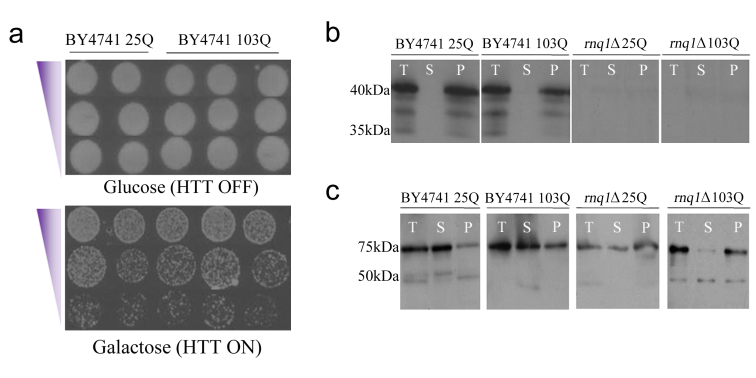
a) Expression of the integrated HTT103Q construct in BY4741 yielded no toxicity or cell death, even when Rnq1p was expressed on a high copy number plasmid, labelled here as 2µ. Equal numbers of cells were serially diluted threefold and plated on medium containing glucose to assess cell numbers and medium containing galactose to induce the expression of mutant HTT. b) [*PIN* ^+^] is present in the integrated BY4741 strains and is absent in the *rnq1*Δ background. (T=total protein, S=soluble protein, and P=insoluble protein). The [PIN] status of the cells was determined by using standard immunoblot techniques and an antibody raised against yeast Rnq1p. c) [*PSI* ^+^] is present in both the integrated BY4741 strains and the *rnq1*Δ background. The presence of [*PSI* ^+^] was assessed using an antibody raised against yeast Sup35p.

The effect of expressing the integrated HTT constructs was assessed via growth assays and fluorescence microscopy. Surprisingly, we observed that expression of HTT103Q in the integrated strain did not impair growth in multiple integrants generated from independent transformations (Figure 1a). As the presence of the [*PIN^+^*] and [*PSI^+^*] prions modulates mutant HTT aggregation and toxicity in baker’s yeast^25^ , we assessed their status in the integrated strains. We observed that Rnq1p is predominantly found as [*PIN^+^*] in the BY4741 integrated strains, and as expected is not present in the *rnq1*Δ strains (Figure 1b). Sup35p (Figure 1c) was found as [*PSI^+^*] in both the BY4741 and *rnq1*Δ integrated strains. These data confirm that lack of [*PIN^+^*] or [*PSI^+^*] is not responsible for the absence of HTT103Q toxicity in the generated strains.

Despite the lack of HTT103Q-dependent toxicity in the newly created integrated strains (Figure 1a), we observed a high level of inclusion body formation in these cells (Figure 2). Indeed, ~90 % of the cells in the integrated HTT103Q strain contained mutant HTT inclusion bodies 12 hours post induction (Figure 2), while in the *rnq1*Δ strain the number of cells containing inclusions is reduced to ~7 %. Reintroduction of a plasmid expressing Rnq1p into the *rnq1*Δ strain restored inclusion body formation to control levels. Thus, this novel integrated yeast model of HD dissociates the toxicity caused by HTT103Q expression from its aggregation and allowed us to further dissect the relationship between prion-like yeast proteins and the dynamics of mutant HTT aggregation without the confounding effects of toxicity.

**Overexpression of Rnq1p increases the aggregation of mutant huntingtin in yeast:**
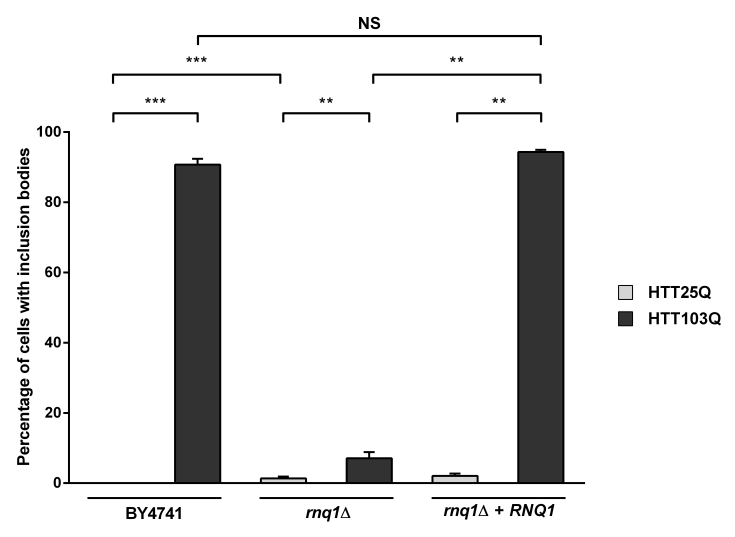
Expression of HTT was induced in overnight cultures and the number of cells containing inclusion bodies was assessed by fluorescence microscopy. Overexpression of Rnq1p in a *rnq1*Δ background restores inclusion body formation in cells expressing HTT103Q. Counts were converted to the percentage of cells containing inclusion bodies and statistical analyses were performed using unpaired, two-way Mann-Whitney tests (**P< 0.01, ***P< 0.001), NS = not significant. All data are shown as the mean ± SEM; n ≥ 5 per genotype.

Interestingly, we found that deletion of the endogenous *RNQ1* gene leads to the formation of inclusion bodies in a small number of cells expressing the HTT25Q construct, which does not normally form these structures (Figure 2; *P*=0.0002). It has recently been observed that a network of proteins in yeast modify the formation of mutant HTT aggregates^26^ , and it is thus possible that deletion of the endogenous *RNQ1* disrupts the balance between other yeast genes that govern HTT aggregation, leading to the aggregation of the HTT25Q construct, which is highly enriched for glutamines in comparison to most endogenous yeast proteins.

**Ybr016wp and Sup35p modulate HTT aggregation independently of Rnq1p: **We next focused on characterizing the role of Ybr016wp and Sup35p in the aggregation dynamics of mutant HTT in yeast. We initially examined the ability of Ybr016wp to modulate the number of cells containing inclusion bodies when overexpressed in the presence and absence of Rnq1p (Figure 3). Interestingly, overexpression of Ybr016wp causes a small but significant increase in the formation of inclusion bodies in HTT25Q-expressing BY4741 (~ 12 %; *P*=0.0022) and *rnq1*Δ (~ 7 %; *P*=0.0007) cells (Figure 3). A possible explanation for this is the high level of Ybr016wp overexpression, which contains five consecutive perfect repeats (GYNQQ) that are enriched for asparagine, glutamine, tyrosine and glycine. Overexpression of such sequences induces aggregation of other proteins, and could seed formation of inclusion bodies by polyQ-rich sequences^22^^,^ 27 . Analysis of the HTT103Q integrated strain revealed that Ybr016wp overexpression yileds a small but significant increase in the number of BY4741 cells exhibiting inclusion bodies (*P*=0.008). Ybr016wp overexpression in the *rnq1*Δ background also significantly increased the number of *rnq1*Δ cells forming HTT103Q inclusions, from ~ 7 % to ~ 16 % (*P*=0.0293). These data indicate that Ybr016wp is able to partially compensate for the loss of Rnq1p in the *rnq1*Δ background with respect to mutant HTT aggregation, and that this aggregation is not polyQ-length dependent.

**Overexpression of Ybr016wp increases the aggregation of mutant huntingtin in yeast:**
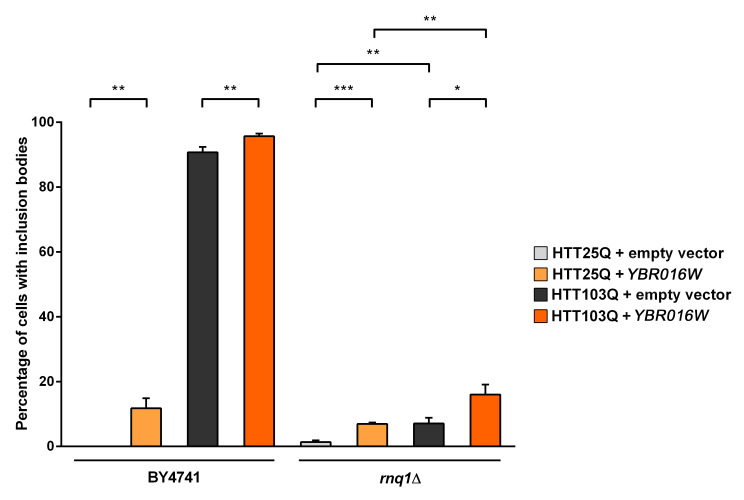
Expression of HTT was induced in overnight cultures and the number of cells containing inclusion bodies was assessed by fluorescence microscopy. Overexpression of Ybr016wp in a*rnq1*Δ background increases inclusion body formation in both HTT25Q and HTT103Q expressing cells. Counts were converted to the percentage of cells containing inclusion bodies and statistical analyses were performed using unpaired, two-way Mann-Whitney tests (*P< 0.05, **P< 0.01, ***P< 0.001). All data are shown as the mean ± SEM; n ≥ 6 per genotype.

As the presence of Rnq1p is crucial for the *de novo* formation of [*PSI^+^*] as well as the formation of mutant HTT aggregates in yeast, we next investigated the connection between Sup35p expression and mutant HTT aggregation by overexpressing Sup35p. As with Ybr016wp, overexpression of Sup35p leads to a small but significant increase in the formation of HTT25Q inclusion bodies in the BY4741 background (Figure 4; *P* < 0.0001). It is possible that these inclusion bodies form due to the presence of [*PSI^+^*] in the cells, as formation of [*PSI^+^*] can be triggered by overexpression of Sup35p^28^ , which is able to cause aggregation of heterogeneous polyQ-rich sequences^29^ . Interestingly, in the *rnq1*Δ strain overexpressing Sup35p the formation of HTT25Q inclusion bodies is dramatically increased (~ 68 %) compared with the parental BY4741 strain (~ 12 %; *P*=0.0264). As mentioned earlier a network of proteins is able to modify aggregation of mutant HTT in yeast^25^ and has also been found to trigger the formation of [*PSI^+^*]. It is therefore possible that this network is activated by the absence of endogenous Rnq1p in the *rnq1*Δ strain, and is further stimulated by the presence of [*PSI^+^*]. Interestingly, the HTT25Q and HTT103Q strains exhibit a similar number of cells with aggregates in the *rnq1*Δ background with Sup35p expression, indicating that this substitution is not polyQ-length dependent, and potentiates the aggregation of shorter polyQ stretches.

**Overexpression of Sup35p increases the aggregation of mutant huntingtin in yeast:**
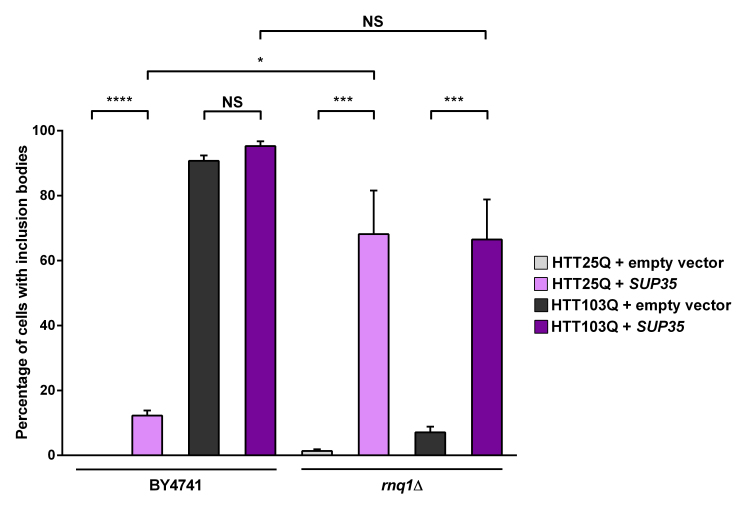
Expression of HTT was induced in overnight cultures and the number of cells containing inclusion bodies was assessed by fluorescence microscopy. Overexpression of Sup35p in a *rnq1*Δ background greatly enhances inclusion body formation in a non-polyglutamine dependent manner. Counts were converted to the percentage of cells containing inclusion bodies and statistical analyses were performed using unpaired, two-way Mann-Whitney tests (*** *P*< 0.001, ****P< 0.0001), NS = not significant. All data are shown as the mean ± SEM; n ≥ 7 per genotype.

## Discussion

Our studies indicate that overexpression of Rnq1p, Sup35p and Ybr016wp can modulate the formation of mutant HTT aggregates in yeast. Furthermore, the induction of mutant HTT inclusion body formation by both Sup35p and Ybr016wp is in part dependent upon the presence of Rnq1p in the cell. The mechanism(s) by which these two aggregation-prone proteins modulate mutant HTT aggregation is not clear, nor is it known if they are acting in a similar manner. In the case of Sup35p it has been proposed that there are two groups of factors that govern both the formation of mutant HTT aggregates and the Sup35p to [*PSI^+^*] switch in yeast^26^ . The first group is involved in the formation of large mutant HTT aggregates and the switch between Sup35p and [*PSI^+^*], while the factors in the second group are crucial for the formation of soluble oligomeric species of mutant HTT, as well as the formation of diffuse [*PSI^+^*] aggregates. These findings further support the hypothesis that yeast prions and glutamine-rich amyloidogenic proteins interact with similar molecular partners upon forming larger amyloid structures in yeast^22^ . Based upon this observation it is possible that mutant HTT interacts directly with Sup35p. Indeed, a recent investigation indicates that mutant HTT can sequester Sup35p in [*PSI^+^*] cells^27^ . As most established yeast prions have a Q/N-rich sequence and mutant HTT can sequester Q-rich sequences in yeast^28^ , it is possible that mutant HTT can sequester other yeast prions. Ybr016wp is poorly characterised, and the relationship between this protein and mutant HTT described here is novel. Although Ybr016wp has an unknown cellular function it contains a CYSTM domain that is characteristic of eukaryotic proteins involved in stress tolerance^30^ and is anchored to the plasma membrane and predicted to be palmitoylated, similar to the human prion protein PrP^C^ 31.

Many highly conserved yeast proteins have prion-like features^22^ , suggesting that human proteins with prion-like domains may behave in a similar manner. Indeed, a recent study has implicated a large number of glutamine and asparagine rich human RNA-binding proteins in the pathology of a range of neurodegenerative diseases^32^. Thus, it is possible that a suite of aggregation prone proteins that modify the aggregation of mutant HTT exists not only in yeast but in humans as well, which may have implications for HD pathogenesis. Indeed, such proteins could modify the aggregation and misfolding of not only mutant HTT, but other amyloidogenic proteins as well, thus informing the development of therapies for HD as well as for other neurodegenerative diseases caused by protein misfolding.
